# Dual Twist Channel Angular Extrusion for Ultrafine-Grained Material Processing as an Advanced Severe Plastic Deformation Technique: A Finite Element Analysis

**DOI:** 10.12688/f1000research.179642.2

**Published:** 2026-07-14

**Authors:** Vikas Ranjan, Sambit Kumar Mohapatra, Sushanta Tripathy, Ratnakar Das

**Affiliations:** 1 School of Mechanical Engineering, Kalinga Institute of Industrial Technology, Bhubaneswar, Odisha, 751024, India; 2 School of Mechanical Engineering, Kalinga Institute of Industrial Technology, Bhubaneswar, Odisha, 751024, India; 3 School of Mechanical Engineering, Kalinga Institute of Industrial Technology, Bhubaneswar, Odisha, 751024, India; 4 Department of Mechanical Engineering, National Institute of Advanced Manufacturing Technology, Ranchi, Jharkhand, 834003, India

**Keywords:** Dual Twist Channel Angular Extrusion (DTCAE); Severe Plastic Deformation (SPD); Finite Element Method (FEM); Ultra Fine-Grained Materials; Equal Channel Angular Pressing (ECAP).

## Abstract

Dual Twist Channel Angular Extrusion (DTCAE), a modified advanced Severe Plastic Deformation (SPD) technique, is investigated through Finite Element Analysis (FEA). The proposed technique is the cumulation of multiple deformations with Equal Channel Angular Pressing (ECAP) to enhance the process efficiency and strain induction. The DTCAE die facilitates a twist of 90°, before the 110° ECAP, a 45° twist after ECAP in the reverse direction, followed by the extrusion. The extrusion at the die exit introduces backpressure and ensures proper material flow in the die. DEFORM-3D finite element simulation software package is utilized for the deformation, flow characteristics, load requirement etc. The results of the DTCAE simulation revealed the homogeneous and higher amount of strain induction in the billet in comparison to single ECAP. Effective strain distribution contours, Load-stroke plot, and velocity vector map of the work material ensure the progressive deformation and homogeneous strain distribution, establishing the effectiveness of the advanced DTCAE process.

## 1. Introduction

In these decades, there is a huge demand for high-strength to light-weight structural materials, which leads to enhance the research on strengthening mechanisms of materials. Out of many strengthening mechanisms, such as precipitation strengthening, solid solution strengthening, dispersion strengthening, etc., Severe Plastic Deformation (SPD) techniques are one of the most promising techniques. ^1– 3^ Notable SPD techniques include High-Pressure Torsion (HPT), ^4, 5^ Accumulative Roll Bonding (ARB), ^6, 7^ Equal Channel Angular Pressing (ECAP), Twist Extrusion (TE), ^8, 9^ and Repetitive Corrugation and Straightening (RCS) ^10, 11^ etc. ECAP has been extensively studied and investigated by industries and academic research institutes due to its simple processing techniques and ability to induce sufficient shear strain in the pressed material. ^12^ Despite its simple and effective processing, it induces heterogeneously distributed strain in a single pass and needs multiple passes to enhance the strain induction and its homogeneous distribution. To overcome the challenges, many researchers have hybridized different SPD techniques to cumulate the deformations in a single die, such as Rotating Backward Extrusion (RBE), Constrained Groove Pressing (CGP), ^13^ Twist extrusion and Incremental HPT (IHPT) etc. All these processes integrate the deformations through multi-axial strain paths to improve uniformity. These methods demonstrate the efficiency of hybrid SPD techniques to overcome the limitations of heterogeneity and multi-pass pressing and facilitate large-scale material processing.

Any SPD processes should meet several requirements: firstly, to obtain UFG structures with prevailing high-angle grain boundaries, secondly, to ensure uniformity in structure distribution, and thirdly, to produce mechanical damage-free, crack-free, or fracture-free specimens. Twist extrusion with non-uniform helical channels was investigated by Valiev et al., ^1^ and revealed that the enhanced shear strain due to cumulation of deformations led to ultrafine-grained (UFG) structures with isotropy in mechanical properties. Applying surface roughening and intermediate annealing to the specimens during SPD significantly enhances the interfacial bonding and suppresses delamination, which results in high ductility and tensile strength. ^14^ Zhilyaev and Langdon ^5^ introduced stepped anvils for HPT to generate functionally graded material structures with enhanced surface hardness and excellent ductility. Moreover, the application of varying rotational speeds and varying axial loading ^4^ has been observed to enhance the structure uniformity and fatigue performance. In CGP, ^13^ refining groove geometry and optimizing the pressing sequence have improvements in tribo-mechanical stability under cyclic loading. These modifications and advancements in process design and deformation strategy can significantly enhance the efficiency, material qualities, and broader utility of hybrid-SPD techniques.

Though all the deformation processes in SPD occur in a closed chamber, to understand the complex deformation behaviour, there is an urge to utilise Finite Element Simulation techniques. ^15, 16^ The detailed analysis of the SPDs, including Load versus stroke plot, maximum strain and its distribution, maximum stress and its distribution, material flow characteristics, die wear, microstructural investigation can be done extensively without any material waste. DEFORM-3D is widely recognised for its robustness and accuracy in modelling all metal forming operations among available platforms. Its ability to simulate intense plastic strains, coupled with features like auto-remeshing, temperature coupling, microstructural modelling, and advanced material modelling, makes it particularly suitable for all SPD studies. ^17, 18^ DEFORM-3D not only reduces the need for costly trial-and-error experiments, it also enhances the process efficiency by optimizing the variable parameters and die profiles with utmost reliable validation.

In this investigation, a novel DTCAE die is introduced for a complex deformation that integrates double twisting, ECAP and extrusion. The effectiveness of the die is analysed by DEFORM-3D finite element simulation. Maximum load requirement, deformation pattern, flow characteristics, strain and temperature distribution were focused in the SPD analysis.

## 2. Die design and function of DTCAE

The die channel was designed to generate bi-directional twisting and shearing forces in the material. To ensure the complete flow of material in the channels, back pressure is essential. To induce the back pressure, the same shape extrusion is provided at the exit of the die. These cumulation of deformation patterns differentiates it from conventional ECAP/ECAE processes ^19, 20^ or multi-angular twist channel extrusion. ^21^

**Figure 1.**
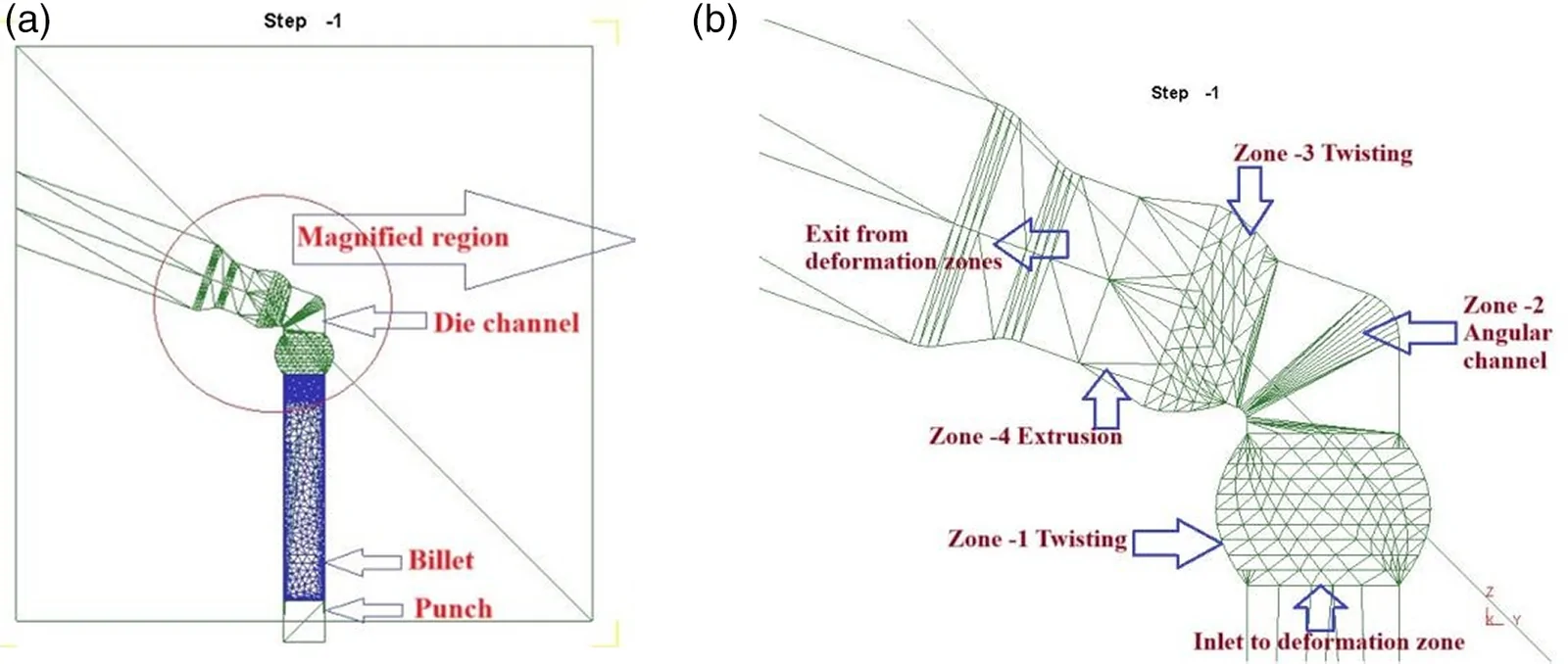
(a) Die setup after proper positioning in the DEFORM-3D platform (b) zone-wise magnified view of the die.

A thorough understanding of die geometry and processing parameters, such as twist angle, ECAP channel angle, length of deformation, friction coefficient, operating temperature, number of processing passes, and punch velocity is important for designing the die to induce homogeneous strain induction at lower extrusion loads. ^22^ The DTCAE die consists of a 110° angular channel ( φ ) and incorporates two twist sections, i.e., 90° clockwise twist before and 45° anticlockwise twist after the channel followed by extrusion as shown in Figure 1. The Figure 1(a) shows the wire frame model of the tooling setup ready for simulation in DEFORM-3D platform, and Figure 1(b) highlights the stages of deformation zone. The four deformation zones of the die are as follows:

***Pre-channel twist (90°):*** During the initial deformation phase (Zone-1), the billet is subjected to a 90° twist in clockwise direction before entering the angular channel. To ensure the uniform torsional deformation, the twist zone length is designed to match the billet’s width.

***Angular Channel (110°):*** After the first tortional deformation, the billet is subjected to an angular channel with a channel angle (Φ) of 110° and an outer corner angle (Ψ) of 20°. ^23^ As presented in Figure 1(b) (Zone-2), the zone is the conventional ECAP and it will impart shear deformation to the pretrained material and generating self-imposed back pressure. ^3^

***Post-Channel Reverse Twist (45°):*** The billet is subjected to a 45° twist in the anti-clockwise direction and reverse to that of Zone-1. The length of this zone remained proportional to the Zone-1, i.e. maintained at half the billet’s width, ensuring effective secondary torsional deformation. This region is identified as Zone-3 and represented in Figure 1(b).

***Extrusion (Zone-4):*** The same shape reduction, characterized by a specific extrusion ratio (R) of 1.25, is imposed on the billet just before the die exit. This region generates self-induced back pressure, thereby increasing resistance to material flow. This region is represented as Zone-4 and illustrated in Figure 1(b).

## 3. Finite element analysis (FEA)

DEFORM-3D, a robust finite element simulation package, was employed for this DTCAE analysis. The rigid-visco-plastic model with an integrated heat transfer function was chosen for this study. The simulation workflow involved the integrated use of DEFORM-3D’s pre-processor, run engine, and post-processing modules. ^24^ The designed die and tooling setup is imported in the STL files format into preprocessor interface. Proper orientation of all components was done with respect to the X, Y, and Z Cartesian coordinate system. Using the master–slave approach (assigning the workpiece as slave and tooling as masters), inter-object relations were defined. Thermo-viscoplastic material model was employed for the billet, while die-tooling setup was modeled as thermo-rigid to capture their distinct behaviors. ^25^ AA-6063 aluminium alloy was chosen as the billet material, and the material properties were considered from the program’s integrated material library. The boundary conditions and assumptions considered in the simulation model are summarized in Table 1.

**Table 1.** Considerations for the numerical simulation.

Input parameters	Values and assumptions
Flow stress	$\overset{-}{\sigma }=\overset{-}{\sigma }(\overset{-}{\varepsilon },\dot{\overset{-}{\varepsilon }},$ *T* )
Mesh type and number of elements	Tetrahedral mesh and 1,00,000
Operating temperature (billet, tooling setup and environment)	20°C
Friction model and amount	Shear friction model and 0.12
Heat transfer coefficient at die billet interface	5 N/sec/mm/C
Convection coefficient	0.02 N/sec/mm/C
Punch velocity	0.05 mm/sec

For the numerical simulation, a Lagrangian incremental simulation model was adopted. The conjugate gradient solver along with the direct iteration technique is adopted to efficiently handle the non-linearities associated with the deformation process. The mesh density considered in this numerical simulation was selected based on our previous work. ^23^ Furthermore, the selected mesh density provides a suitable balance between solution accuracy and computational efficiency. The global remeshing option was adopted to maintain proper mesh quality during severe deformation. To trigger the remeshing when element distortion exceeded the threshold, a relative interface depth of 0.7 was adopted. ^20^ The considered friction coefficient was taken from the software database for the material and operating temperature condition. These selection of parameters facilitated accurate tracking of material flow, stress-strain and temperature evolution throughout the DTCAE process.

## 4. Results and discussion

### 4.1 Material flow characteristics in DTCAE

The flow characteristics of the material are directly related to the load requirement and strain induction. In DTCAE, material flow is governed by sequential die-induced deformations. A 90° clockwise twist generates shear at and multidirectional strain. The strain at the core of the billet is observed less. The deformation through 110° ECAP channel imposes plane strain. The 45° anticlockwise twist that introduces cross-shear, ensuring homogeneous strain distribution and activation of multiple slip systems. Just before the die exit, the billet is extruded “R = 1.25”, causing dimensional reduction and microstructural elongation with surface deformations. The cumulation of forward and reverse torsion, angular channel pressing followed by extrusion promotes uniform flow and homogeneous deformation across the billet.

**Figure 2.**
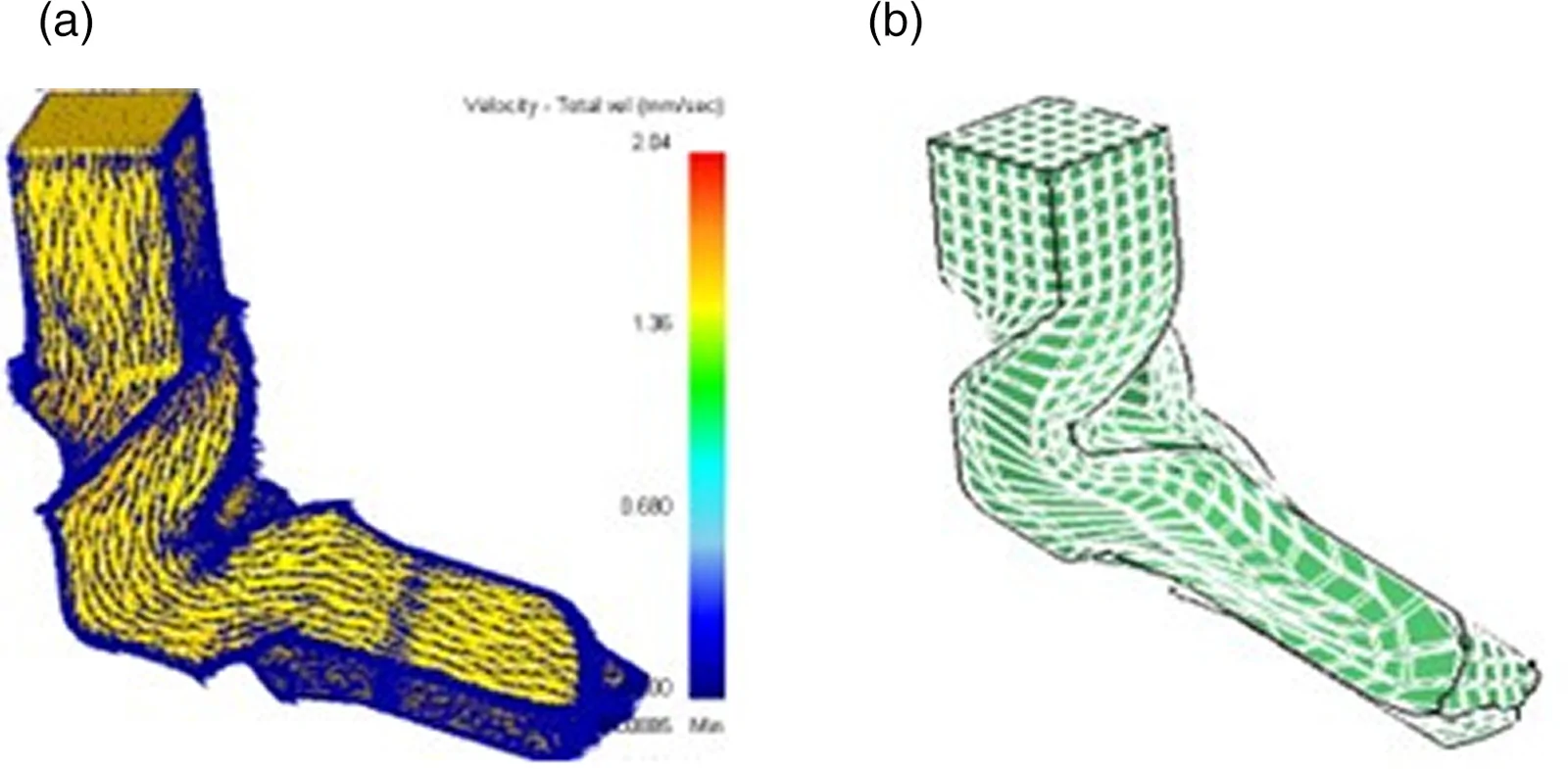
Material Flow characteristics in DTCAE: (a) velocity vector distribution and (b) distorted grid pattern indicating deformation behaviour.

This flow pattern is depicted in both the velocity vector diagram and three dimensional grid pattern in Figure 2(a) and (b), respectively. The velocity vector diagram reveals smooth material flow through each deformation zone, while grid pattern analysis reveals the idea of strain distribution across the billet, assuring homogeneous deformation and demonstrating the die geometry’s effectiveness in promoting consistent flow.

### 4.2 Effective strain distribution

**Figure 3.**
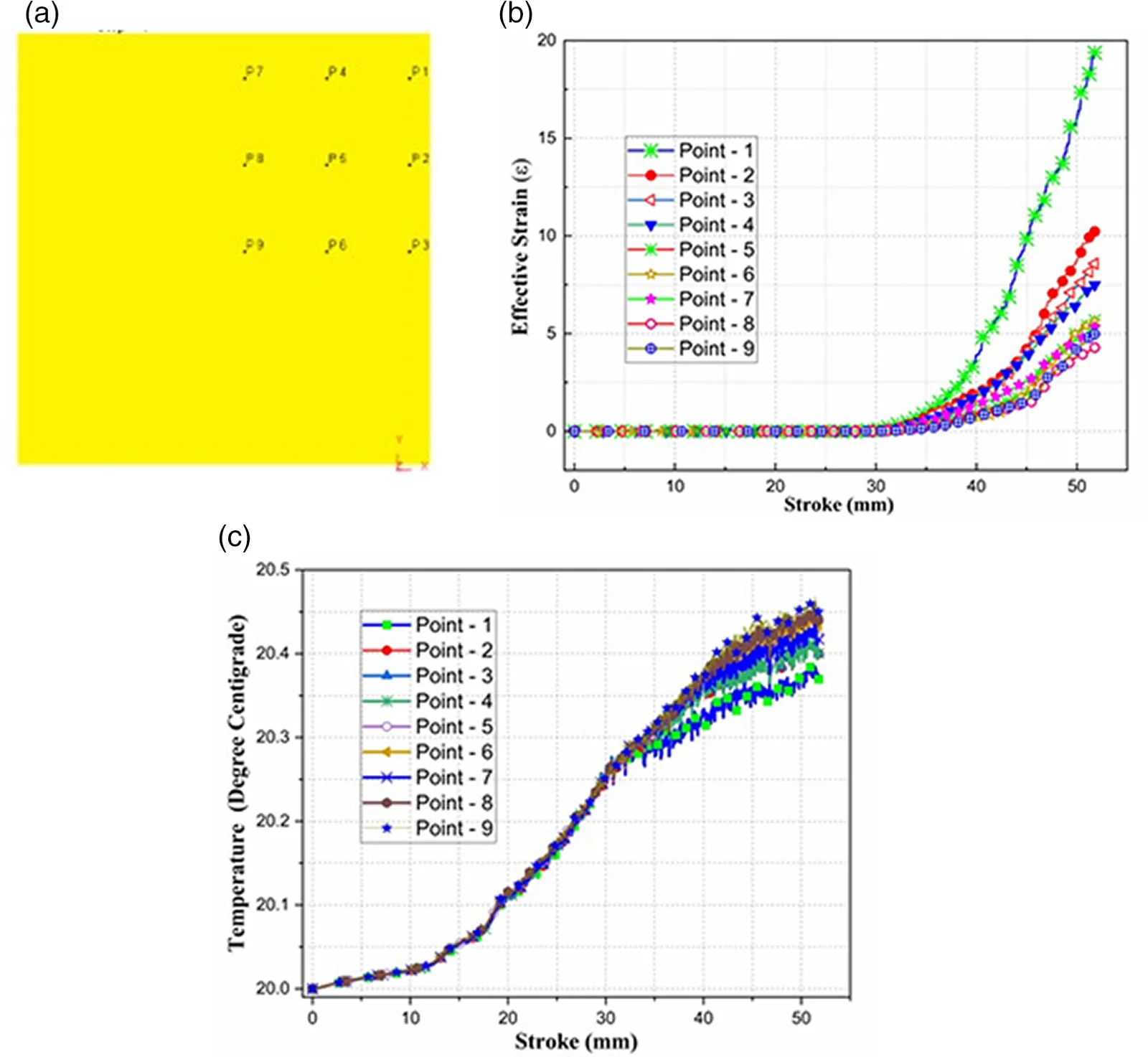
(a) Nine tracking points in one quadrant (b) Strain and (c) Temperature distribution.

To capture the temperature, strain and velocity distribution across the billet during the DTCAE process, nine tracking points at the billet cross-section were strategically placed at symmetric positions as illustrated in Figure 3(a). To reflect both surface and core deformation behaviors these points were distributed at the edge, mid-edge, and central regions in one quadrant zone.

The effective strain induction in the DTCAE process was evaluated through the selected nine tracking points. The effective strain vs stroke for all nine points is plotted and presented in Figure 3(b), indicating a progressive rise in effective strain with stroke as it starts passing through the deformation zones. ^26– 28^ The outer surface is subjected to huge strain induction.

Point 1, as in Figure 3(a), located near the outer corner of the billet, exhibited the highest strain, approximately 18, while the central point, i.e., point 9, recorded the lowest value, around 4. The dual and opposite twist channels in DTCAE introduce additional shear planes, leading to more complex and multidirectional material flow compared to conventional SPDs. This enhanced deformation technique not only enhances the net strain accumulation per pass but also promotes UFG refinement and microstructural evolution. Thus, DTCAE demonstrates a distinct advantage over TE, ECAP, RCS etc. by inducing higher strain intensities in a single pass. The critical deformation zones make it a more effective SPD technique for advanced material processing.

The temperature distribution in the specimen in DTCAE process was estimated and illustrated in Figure 3(c) from the FEA. The initial billet temperature was set 20°C. The simulation adopted a very low convection coefficient of 0.02 N/s-mm-°C and a moderate conduction coefficient of 5 N/s-mm-°C to replicate approximately-isothermal conditions. The temperature profile at nine selected points is traced and found a minimal rise from 20.0°C to approximately 20.45°C over the stroke length. The minimal temperature variation, with the least rise at the boundary and the highest at the core, confirms the accuracy and physical consistency of the model. This negligible thermal gradient confirms the quasi-isothermal deformation of the work material in the present setup. This temperature distribution indicates the realistic heat generation in the severe plastic deformation considering the assumed thermal boundary conditions.

### 4.3 Effect of dual twist and extrusion zone

The dual twist configuration appears to significantly improve strain homogeneity by disrupting unidirectional material flow and reorienting the flow at different critical zones. This not only enhances local plasticity but also promotes activation of multiple slip systems, even at the core region, which reportedly shows lower strain accumulation in single-mode SPD techniques. The geometric reduction due to the extrusion, contributes to a gradual development of back pressure, ensuring the complete full flow, aiding in consistent deformation across the billet. The velocity contour plot depicts an even transition of material flow through the die channel without formation of dead zones or localised turbulence, which confirms the effectiveness of twist-extrusion. These cumulate effects make DTCAE highly efficient for UFG structure in single pass while minimizing internal defects and enhancing material properties.

### 4.3 Load-displacement curve

**Figure 4.**
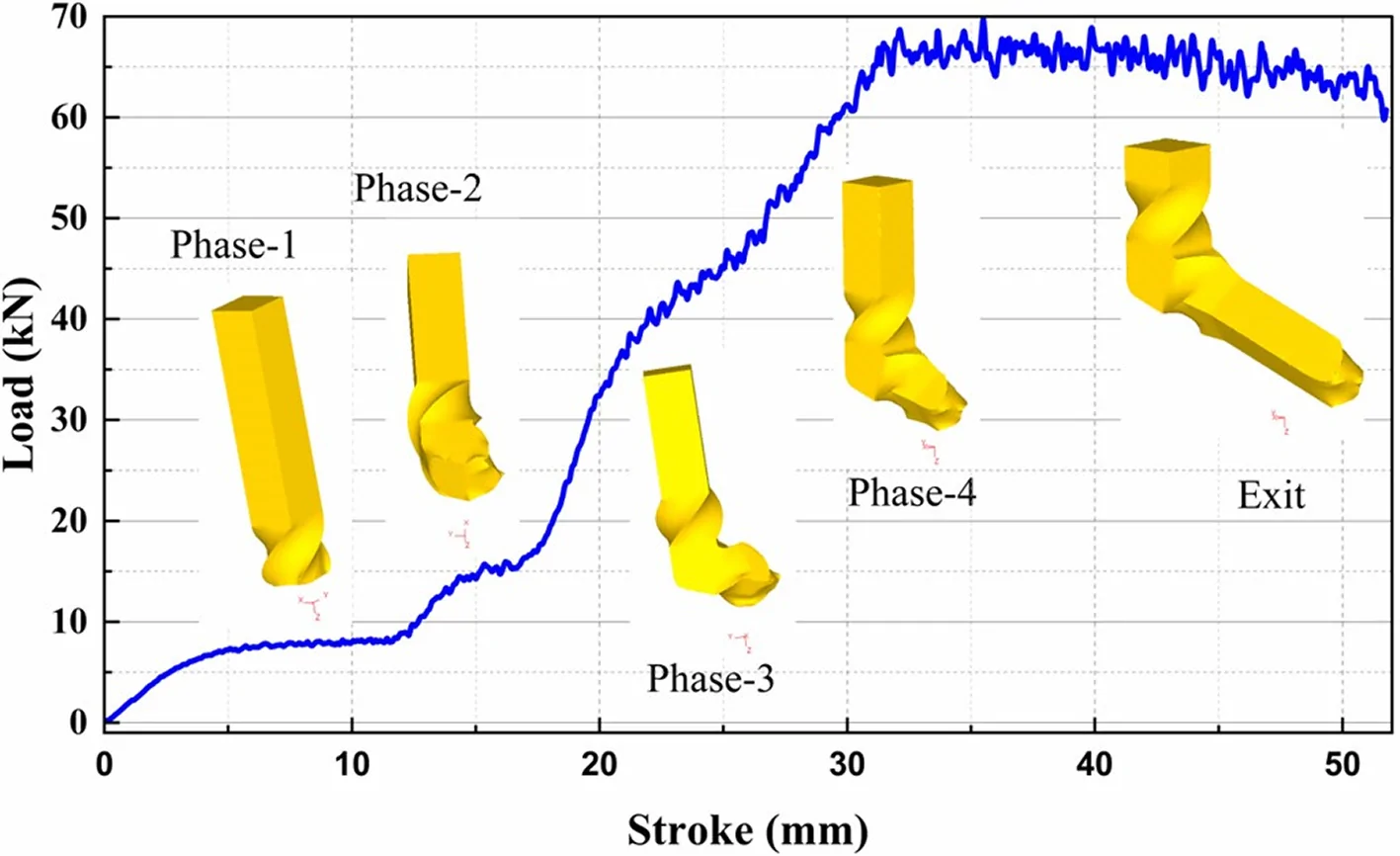
Load vs stroke with the stages of deformation.

Figure 4 illustrates the Load vs. Stroke plot obtained from the DEFORM-3D simulation of the DTCAE process. The plot provides detailed insights into the material flow, maximum load requirement and deformation behaviour throughout the deformation cycle. At the beginning stage, corresponding to Zone-1, the load increases gradually with stroke. In this stage, the billet undergoes Phase-1 deformation ‘a 90° twisting’. Up to approximately 11 mm of stroke length, is characterised by increasing resistance due to plastic deformation and die-billet interfacial friction.

Between approximately 11 mm and 23 mm of stroke, a notable rise in load is observed, i.e., Phase-2 deformation, indicating maximum forming resistance as the billet experiences shear deformation within the angular channel for ECAP.

Subsequently, in Phase-3 deformation, i.e., within around 23 mm to 28 mm of stroke, corresponding to a reverse twisting of 45°, a visible increase in load occurs. This significant rise in load is attributed to material reorientation, additional shear strain generation and activation of multiple slip systems.

In Zone IV, a further rise in load is observed during Phase-4 deformation, i.e., between approximately 28 mm and 32 mm of stroke. This rise is mainly due to cumulative strain hardening effect of the billet material and increased resistance due to extrusion at the die exit region.

At the end, the billet exits the die, as illustrated. The process needs a peak load of around 70 kN, corresponding to the billet’s complete traversal through all the complex die channels, demonstrating the material’s deformation response in DTCAE process.

## 5. Conclusion

The finite element investigation of the Dual Twist Channel Angular Extrusion (DTCAE) process was conducted successfully through DEFORM-3D software package. With significant strain accumulation, moderate and uniform temperature rise, and distinct deformation phases, the process demonstrates its superiority as an effective and efficient Severe Plastic Deformation (SPD) technique. Effective strain distribution analysis through the point tracking method reveals the highest strain accumulations near outer deformation zones, and the lowest at the core zone. The core zone strain induction indicates enhanced plastic deformation due to complex channel geometry and ensures the process supremacy over other SPDs. The load–stroke plot distinctly marks four deformation phases, capturing the variation in resistance as the billet flows through complex channels. These results corroborate that DTCAE not only refines material through effective strain by inducing multiple slip systems but also ensures its feasibility, making it a robust and scalable alternative to conventional SPD methods. Future work on this technique may focus on extending the experimentation and comparison of results for a more comprehensive analysis.

## Data Availability

All data supporting the findings of this study, including raw and processed data, are openly available in Figshare under a CC-BY 4.0 license and can be accessed via. DOI: https://doi.org/10.6084/m9.figshare.31879312. ^29^ **Repository name:** *Finite Element Simulation Dataset for DTCAE Process*. https://doi.org/10.6084/m9.figshare.31879312. ^29^ The project contains the following underlying data: Underlying-RAW-Fig 3. (c) Temperature Distribution.xlsx (the Raw data of Fig 3. (c) from which the graph plot is made). Underlying-RAW-Fig 4. Load vs stroke.xlsx (the Raw data Fig 4 from which the graph plot is made). Underlying-RAW-Fig 3. (b) strain.xlsx (the Raw data Fig 3. (b) from which the graph plot is made). Underlying- Fig 1. (a).jpg (the figure presented in Fig 1. (a)). Underlying- Fig 1. (b).jpg (the figure presented in Fig 1. (b)). Underlying- Fig 2. (a).jpg (the figure presented in Fig 2. (a)). Underlying- Fig 2. (b).jpg (the figure presented in Fig 2. (b)). Underlying- Fig 3. (a).jpg (the figure presented in Fig 3. (a)). Underlying- Fig 3. (b).jpg (the figure presented in Fig 3. (b)). Underlying- Fig 3. (c).jpg (the figure presented in Fig 3. (c)). Underlying- Fig 4.jpg (the figure presented in Fig 4). **Repository name:** *Finite Element Simulation Dataset for DTCAE Process*. https://doi.org/10.6084/m9.figshare.31879312. ^29^ The project contains the following underlying data: DTCAE_Simulation Higher_mesh (it is the key file of the simulation and will only open in DEFORM-3D software). EXTENDED-RAW-Assumptions (some of the assumptions there in Table −1 and others are there which may help others during simulation considerations).
