# Supplementary material for: Dual Twist Channel Angular Extrusion for Ultrafine-Grained Material Processing as an Advanced Severe Plastic Deformation Technique: A Finite Element Analysis
Source: F1000Res. 2026 Jul 14;15:679. Originally published 2026 May 8. [Version 2] doi: 10.12688/f1000research.179642.2 (PMC13198697; doi:10.12688/f1000research.179642.2)
Supplement: Peer review history

# Reviewer report: Reviewer response for version 1

**DOI:** 10.5256/f1000research.198176.r483638

**Published:** 2026-05-23

**Authors:** Akhtar Khan

**Affiliations:** 1 IIIT Kurnool, Kurnool, India

Dear Authors,

 I recommend/approve the manuscript entitled “Dual Twist Channel Angular Extrusion for Ultrafine-Grained Material Processing as an Advanced Severe Plastic Deformation Technique: A Finite Element Analysis” for indexing in its present form, as the study makes a novel and relevant contribution to the field of Severe Plastic Deformation (SPD) processing. The proposed DTCAE methodology appears promising for achieving improved strain distribution and ultrafine-grain refinement via advanced deformation pathways.

 Some suggestions are listed below:

 1.    The justification for the selected friction coefficient/value used in the finite element simulation has not been clearly explained. The authors are encouraged to provide a clear rationale or supporting literature for the considered friction conditions. 

 2.    Although the numerical investigation is informative, experimental validation of the DTCAE process would significantly strengthen the findings. The authors may include this aspect as part of future research.

Is the work clearly and accurately presented and does it cite the current literature?

Yes

If applicable, is the statistical analysis and its interpretation appropriate?

Yes

Are all the source data underlying the results available to ensure full reproducibility?

Yes

Is the study design appropriate and is the work technically sound?

Yes

Are the conclusions drawn adequately supported by the results?

Yes

Are sufficient details of methods and analysis provided to allow replication by others?

Yes

Reviewer Expertise:

Manufacturing Science and Engineering

I confirm that I have read this submission and believe that I have an appropriate level of expertise to confirm that it is of an acceptable scientific standard.

# Response

**Published:** 2026-06-20

**Authors:** Dr. Sambit Kumar Mohapatra

**Affiliations:** School of Mechanical Engineeri, Kalinga Institute of Industrial Technology School of Mechanical Engineering, Bhubaneswar, Odisha, India

Response 1: The friction conditions were considered as per the suggestion of the database of the software, and the same statement is added in the article.

 Response 2: Thank you for the suggestion. Future scope and opportunity were added in the conclusion.

# Reviewer report: Reviewer response for version 1

**DOI:** 10.5256/f1000research.198176.r483632

**Published:** 2026-05-13

**Authors:** Suraj Kumar Behera

**Affiliations:** 1 National Institute of Technology Rourkela, Rourkela, Odisha, India

The manuscript entitled “Dual Twist Channel Angular Extrusion for Ultrafine-Grained Material Processing as an Advanced Severe Plastic Deformation Technique: A Finite Element Analysis” is recommended for the indexing in its current form, considering the novelty and relevance of the proposed DTCAE approach in the field of Severe Plastic Deformation (SPD). The study provides useful insights into strain behaviour and deformation characteristics through finite element analysis, and the proposed technique appears to be a promising advancement for ultrafine-grained material processing.

 The following suggestions may further improve the manuscript:

1. The paper uses finite numbers of tetrahedral elements but does not justify mesh independence. The confirmation or convergence test for the selected mesh size may be included or properly referred to in order to ensure the accuracy and reliability of the simulation results.
2. Page 4: tortional should be torsional
3. Page 3: 90°clockwise twist before and 45°anticlockwise , there should be a gap between numbers and texts.
4. Page 7: instead of “huge rise in load”, author may write “significant rise in load”
5. Figure 2 can be more descriptive and written as “Fig. 2. Material flow characteristics in DTCAE: (a) velocity vector distribution and (b) distorted grid pattern indicating deformation behaviour.”
6. Flow Stress in Table 1, The equation can be re-written for better readability.
7. References 1 and 14 are identical similarly References 5 and 16 are also identical. These duplicates should be removed and numbering updated
8. Current conclusion is acceptable but can be strengthened with future work.
9. As a future scope of the work, simulations at elevated processing temperatures may be conducted to evaluate the deformation behavior and strain distribution under thermally assisted conditions.

Is the work clearly and accurately presented and does it cite the current literature?

Yes

If applicable, is the statistical analysis and its interpretation appropriate?

Yes

Are all the source data underlying the results available to ensure full reproducibility?

Yes

Is the study design appropriate and is the work technically sound?

Yes

Are the conclusions drawn adequately supported by the results?

Yes

Are sufficient details of methods and analysis provided to allow replication by others?

Yes

Reviewer Expertise:

Finite Element Analysis, Rotordynamics, Cryogenics

I confirm that I have read this submission and believe that I have an appropriate level of expertise to confirm that it is of an acceptable scientific standard.

# Response

**Published:** 2026-06-20

**Authors:** Dr. Sambit Kumar Mohapatra

**Affiliations:** School of Mechanical Engineeri, Kalinga Institute of Industrial Technology School of Mechanical Engineering, Bhubaneswar, Odisha, India

Response 1: The authors thank the reviewer for the valuable comment. The mesh configuration adopted in the present study was based on our previously validated finite element model, where the numerical predictions showed good agreement with experimental results. Therefore, the same meshing strategy was employed in the current work to ensure reliable simulation outcomes. This was highlighted and properly cited.

 Response 2: Thank you for the observation. It was modified.

 Response 3: Thank you for the observation. It was modified and space was provided in page number 3.

 Response 4: Thank you for the suggestion. It was modified.

 Response 5: Thank you for the suggestion. It was modified.

 Response 6: Thank you for the suggestion. It was modified.

 Response 7: The authors thank the reviewer for the observation. The duplicate references have been identified and removed. The corresponding reference numbering will be updated in the final revised version during the production/typesetting process.

 Response 8: Thank you for the suggestion. The conclusion is strengthened by adding future scope and opportunity.

 Response 9: Thank you for the suggestion. Future scope and opportunity were added in the conclusion.
